# The effect of severe intensity bouts on muscle oxygen saturation responses in trained cyclists

**DOI:** 10.3389/fspor.2023.1086227

**Published:** 2023-02-23

**Authors:** Assaf Yogev, Jem Arnold, Hannah Nelson, David C. Clarke, Jordan A. Guenette, Ben C. Sporer, Michael S. Koehle

**Affiliations:** ^1^ Environmental Physiology Laboratory, The University of British Columbia, School of Kinesiology, Vancouver, BC, Canada; ^2^ Department of Biomedical Physiology and Kinesiology and Sports Analytics Group, Simon Fraser University, Burnaby, Canada; ^3^ Deptartment of Physical Therapy, The University of British Columbia, Vancouver, BC, Canada; ^4^ Centre for Heart Lung Innovation, Providence Research, The University of British Columbia and St. Paul's Hospital, Vancouver, BC, Canada; ^5^ Department of Family Practice, Vancouver Whitecaps FC, Vancouver, BC, Canada; ^6^ Division of Sports Medicine, The University of British Columbia, Vancouver, BC, Canada

**Keywords:** muscle oxygenation, near-infrared spectroscopy, cycling, severe intensity bouts, wearable, exercise, exercise testing, cardiorespiratory fitness

## Abstract

Near-infrared spectroscopy (NIRS) quantifies muscle oxygenation (SmO_2_) during exercise. Muscle oxygenation response to self-paced, severe-intensity cycling remains unclear. Observing SmO_2_ can provide cycling professionals with the ability to assess muscular response, helping optimize decision-making. We aimed to describe the effect of self-paced severe intensity bouts on SmO_2_, measured noninvasively by a wearable NIRS sensor on the vastus lateralis (VL) muscle, and examine its reliability. We hypothesized a greater desaturation response with each bout, whereas, between trials, good reliability would be observed. Fourteen recreationally trained, and trained cyclists completed a ramp test to determine the power output (PO) at the respiratory compensation point (RCP). Athletes completed two subsequent visits of 50-minute sessions that included four severe-intensity bouts done at 5% above RCP PO. Muscle oxygenation in the VL was monitored using a wearable NIRS device. Measures included mean PO, heart-rate (HR), cadence, and SmO_2_ at bout onset, during work (work SmO_2_), and *Δ*SmO_2_. The bouts were compared using a one-way repeated measures ANOVA. For significant differences, a Fisher's least square difference *post-hoc* analysis was used. A two-way repeated measures ANOVA was used using trial and bout as main factors. Intraclass correlations (ICC) were used to quantify relative reliability for mean work, and standard error of the measurement (SEM) was used to quantify absolute agreement of mean work SmO_2_. Both PO and cadence showed no effect of bout or trial. Heart-rate at bout 2 (168 ± 8 bpm) and 4 (170 ± 7 bpm) were higher than bout 1 (160 ± 6 bpm). Onset SmO_2_ (%) response significantly increased in the final two bouts of the session. Mean work SmO_2_ increased across bouts, with the highest value displayed in bout 4 (36 ± 22%). *Δ*SmO_2_ showed a smaller desaturation response during bout 4 (27 ± 10%) compared to bout 3 (31 ± 10%). Mean work SmO_2_ ICC showed good reliability (ICC = 0.87), and SEM was 12% (CI 9-15%). We concluded that a non-invasive, affordable, wearable NIRS sensor demonstrated the heterogeneous muscle oxygenation response during severe intensity cycling bouts with good reliability in trained cyclists.

## 1. Introduction

Numerous technological advancements in the sport of cycling provide a unique platform for gathering valuable input regarding the effects of exercise intensity on systemic responses, both in the field and in real-time (1). Such advancements primarily focus on measuring external load during training *via* onboard power meters, which measure power output (PO) directly from the bicycle drivetrain. Additionally, heart rate (HR) measurements are equally popular and provide a useful signal for cardiovascular responses and adaptations (2). Understanding this relationship solely through the lens of HR response provides little information about local intramuscular responses during exercise, especially under severe intensity exercise loads where a non-steady state is expected. Understanding muscle metabolic activity during severe intensity cycling exercise, in conjunction with PO and HR measurements, can provide critical information concerning muscular limitations, which can aid in prescribing specific interventions to optimize cycling performance.

Muscle oxygenation *via* near-infrared spectroscopy (NIRS) has been investigated extensively at a range of exercise intensities and activity types (3). With its rise in popularity, NIRS technology has generated good reproducible results under controlled conditions, primarily with the use of stationary, expensive, laser-based spectrometers (4–7). More recently, continuous-wave light-emitting diode spectrometers offer the possibility of integrating NIRS into portable, wearable units (3). Such designs introduced the possibility of assessing the muscle oxygenation response in training and competition environments, at a greatly reduced cost (3, 8).

Near-infrared spectroscopy is sensitive to the reflectivity of hemoglobin and myoglobin in microcirculation and muscle tissue, detecting their oxygenated (O_2_HbMb) and deoxygenated (HHbMb) forms and their sum total (tHbMb) (9). The relative portion of O_2_Hb + Mb from tHb + Mb is used to estimate percent muscle oxygen saturation (SmO_2_). The latter is commonly used by commercially available wearable NIRS manufacturers (10, 11). SmO_2_ tends to decrease with increasing exercise intensity (12). Multiple studies that measure muscle oxygenation during exercise to task intolerance report attainment of an SmO_2_ nadir at the primary locomotor muscles (13–16). During constant workload exercise, the profile of SmO_2_ is generally the inverse of pulmonary oxygen consumption (17). During severe intensity exercise, we might then expect to see progressive deoxygenation in the SmO_2_ response, similar to the V˙O_2_ slow component (17, 18). The ability to display local SmO_2_ response during submaximal, severe intensities in real-time can assist athletes and performance specialists to track muscle oxygen utilization relative to workload and optimize pacing strategy accordingly. Observing this effect during self-paced, severe intensity bouts during cycling remains undescribed.

Currently, test-retest reliability of NIRS has been investigated at rest and in various exercise contexts (5, 10, 13). For wearable NIRS, assessment of its reliability for measuring muscle oxygenation during severe intensity exercise in field-relevant training environments is needed to reveal possible measurement limitations prior to deployment in the field. Accordingly, the purpose of this study was two-fold: 1) describe the effect of self-paced, severe-intensity cycling bouts on SmO_2_ of the vastus lateralis (VL) muscle and 2) quantify the test-retest reliability of SmO_2_ between cycling exercise sessions. Within session, we hypothesized a decreased SmO_2_ with each bout number, whereas we hypothesized good reliability for SmO_2._

## 2. Materials and methods

### 2.1. Subjects

Fourteen trained, and recreationally trained cyclists (7 females & 7 males, 74.1 ± 10.5 kg, 32.1 ± 7.6 years of age, 170.0 ± 11.0 cm, 11.3 ± 5.4 mm VL skinfold thickness, and 55.0 ± 9.1 ml·kg·min^−1^ maximum oxygen uptake) volunteered and provided written informed consent to participate in the study (19). To obtain sufficient power of *β* = 0.8 with *α* = 0.05, an *a priori* sample size calculation was made in G*Power software (version 3.1.9.7, Kiel, Germany) using previously reported data from other groups that compared SmO_2_ values within and between sessions during ramp incremental tests and severe intensity efforts (16, 20–23). This study was approved by the research ethics committee of The University of British Columbia and was conducted in accordance with principles established in the Declaration of Helsinki, except for registration in a database.

### 2.2. Experimental design

#### 2.2.1. Incremental ramp cycling exercise

The study consisted of three visits. The protocol and method used to estimate PO at the respiratory compensation point (RCP) during the first visit was previously described (15), and included an incremental ramp cycling test from rest to task intolerance. Participants completed the protocol on an electronically controlled, stationary bicycle trainer (KICKR, Wahoo Fitness Inc., Atlanta, GA, USA) using their own bicycle at a gear ratio that simulated their regular indoor training. The ramp rate increased by 1 W every 2 s (30 W·min^−1^), with task intolerance determined as the point at which the participant's self-selected cadence went down by more than 10 revolutions per minute (rpm). Resistance was controlled in ergometer mode using PerfPRO Studio Software^©^ (Hartware Technologies, Rockford, MI, USA). During the exercise test, ventilation (Ve), pulmonary oxygen (V˙O_2_), and carbon dioxide uptake (V˙CO_2_) were measured with an open-circuit expired-gas analysis system (TrueOne 2400; ParvoMedics, Inc, Sandy, UT). Following the test, the PO at the RCP was estimated, correcting for individual muscle to lung transit time and used to calculate the PO during the follow-up sessions (105% of RCP PO) (15).

Pulmonary oxygen uptake (V˙O_2_) was measured with an open-circuit expired-gas analysis system (TrueOne 2400; ParvoMedics, Inc, Sandy, UT). V˙O_2_ data were averaged to 15 s and interpolated to 1 Hz for analysis. V˙O_2_peak was considered the highest average 30-second measurement. The RCP was determined at the point of deflection of V˙_E_ relative to V˙CO_2_, and the second deflection of V˙_E_ relative to V˙O_2_ (24, 25).

An individual mean response time (MRT) representing the delay between muscular metabolic activity and pulmonary response was determined using a recently described protocol (26, 27). Briefly, the subjects performed a baseline warm-up for 6 min at a moderate PO of either 110 W (females) or 140 W (males). Average baseline V˙O_2_ was determined from the final 2 min of the baseline step. The ramp exercise test began with 4 min at 70 W (females) or 100 W (males), before the continuous ramp commenced at 1 W per 2 s. The subject's V˙O_2_ response during the ramp test was compared to their average baseline V˙O_2_. The difference in the instantaneous PO that elicited the same V˙O_2_ response was used to determine the MRT in watts and in seconds. The MRT was then used to shift PO relative to V˙O_2_ for estimation of the PO that elicited RCP (15).

#### 2.2.2. Severe-intensity interval exercise

**Figure 1:**
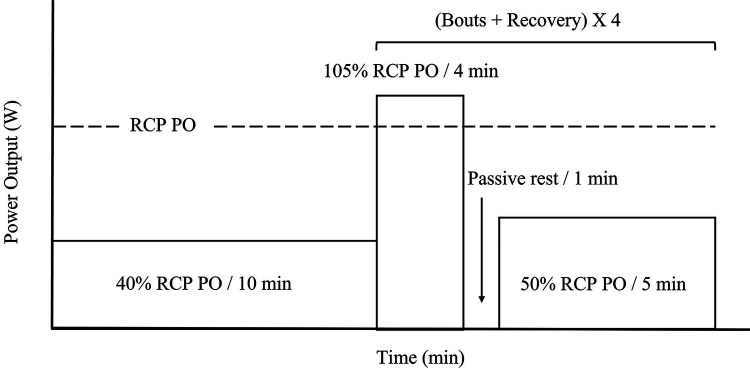
Severe intensity interval exercise protocol. Percent power output (PO) is calculated from PO detected at respiratory compensation point (RCP) (dotted line) from initial ramp test. Ten-minutes warm up at 40% RCP PO, followed by four severe intensity bouts of four minutes at 105% of RCP PO, one minute passive rest, and five minutes active recovery at 50% RCP PO.

The second and third visits included the same ergometer setup as described in the initial visit, and the protocol is shown in Figure 1. The 2nd and 3rd visits are identical and used to determine repeatability. To ensure participants rode within their individual severe intensity domain, bouts were done at 5% above estimated RCP PO from the first visitation (14). The duration of the severe intensity bout was 4 min. The time of the step was selected to allow for a physiological pseudo steady state to occur (28, 29). During both sessions, an initial 10-minute warm-up at 40% of RCP PO was used. Immediately after the warmup, a zero-offset calibration was conducted to ensure the trainer provided accurate resistance. During the first interval session, participants were asked to pedal at a self-selected cadence. Participants were instructed to keep the same cadence (± 5 rpm) and gear ratio to ensure the same workload for the subsequent bouts across both sessions. The use of self-pacing with a target PO, rather than controlling the resistance of the cycling ergometer, was to simulate real-world training conditions. Between bouts, one-minute passive recovery was performed, followed by five minutes at 50% RCP PO. The electronic trainer was set to simulate a gradient of 1%. Measures of PO were measured in all participants, whereas HR and cadence were measure in only 7 participants. These variables were measured continuously using the stationary bicycle trainer, the participant cadence sensor, and a HR monitor (HRM1G; Garmin Ltd., Olathe, KS, USA), respectively.

### 2.3. NIRS

Two wearable NIRS sensors (Moxy Monitor, Fortiori Design LLC., Hutchinson, USA) were used during the test. The Moxy monitor employs four wavelengths of near-infrared light (680, 720, 760, and 800 nm), with source detector separation of 12.5 and 25 mm (10). The sensors were placed on the right and left VL, and the right side was used for analysis and the left was used in case of right sensor failure. In all but one participant, the right sensor was used. The anatomical location on the VL was 1/3 the distance from the proximal pole of the patella to the greater trochanter. Left and right sensors were held in place by the participants' elastic cycling shorts, and both sensors were covered using a light shield supplied by the manufacturer to minimize ultraviolet light interference.

During instrumentation, skinfold thickness was measured and recorded from the right VL with a Harpenden skinfold caliper (Creative Health, Dallas, USA). According to the manufacturer, the Moxy sensor does not require calibration (10, 11). Prior to each trial, the sensor was charged, and both the emitter and receiving optodes were cleaned.

### 2.4. Data analysis

The Moxy sensor provides measures of total heme concentration (tHb + Mb in arbitrary units), and muscle O_2_ saturation (SmO_2_ as a percent). The SmO_2_ signal was used as the primary output variable in this study (10, 11, 21). SmO_2_ was measured every 2 s (0.5 Hz) and raw data were smoothed to 5-second moving averages as per manufacturer default settings. The signal profile was assessed at two points during each of the four severe intensity bouts completed in each session: peak SmO_2_ value at exercise onset and mean 30-sec SmO_2_ at the end of the work phase (work SmO_2_). Additionally, the amplitude of deoxygenation during work was analysed as the change from onset to work SmO_2_ (*Δ*SmO_2_).

**Figure 2:**
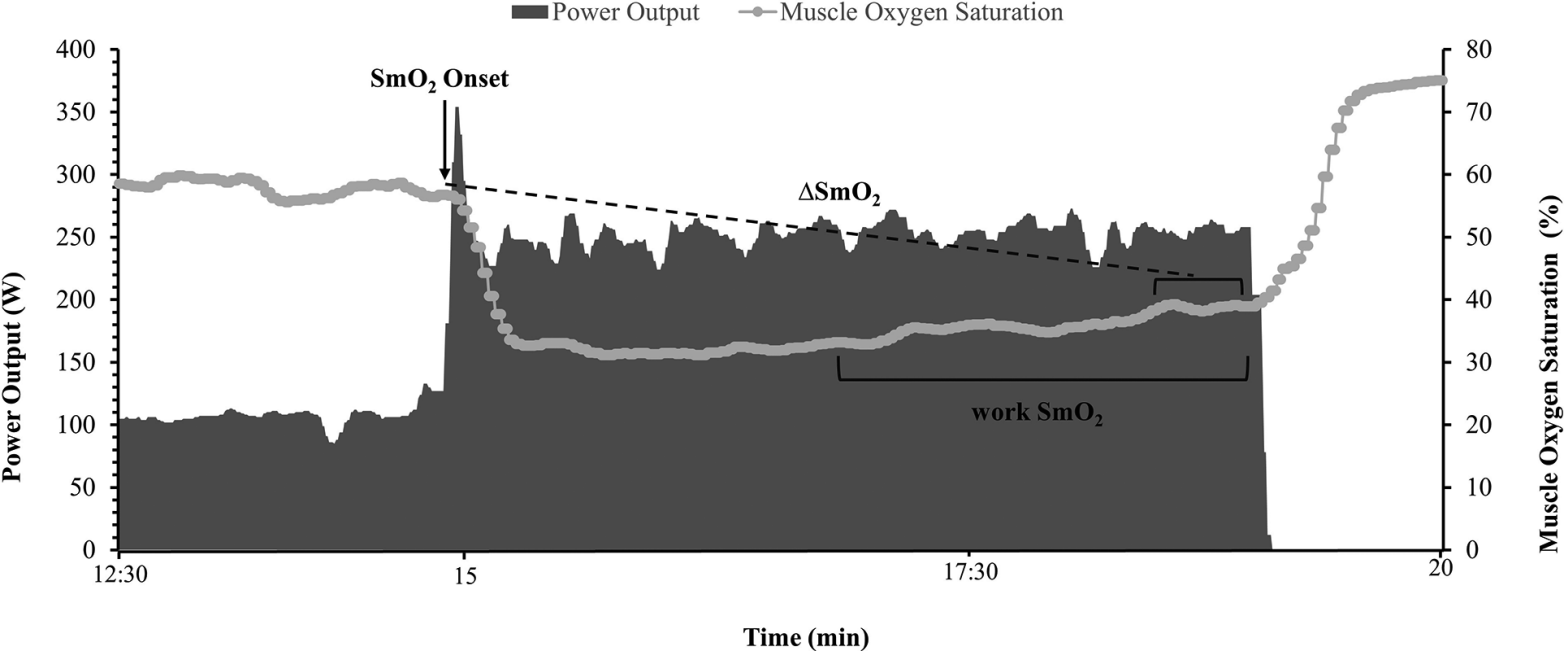
Illustration of muscle oxygen saturation (SmO_2_) at exercise onset (black arrow), mean work phase SmO_2_ during the final two minutes of the severe intensity bout, and *Δ*SmO_2_ calculation of the difference between SmO_2_ at bout onset and the 30s average SmO_2_ at the end of the effort (dotted line).

Data acquisition was done in a training analysis software WKO5 (TrainingPeaks, LLC, Boulder, CO, USA) to find the relevant values at the two phases of each interval. Onset was calculated as the highest SmO_2_ value during the first 60 s of each severe intensity bout. Work SmO_2_ was measured as the mean SmO_2_ of the final two minutes of each bout (Figure 2). The *Δ*SmO_2_ was calculated from the difference between maximum SmO_2_ value at exercise onset to the final 30 s average SmO_2_ of each bout (Figure 2).

### 2.5. Statistical analysis

Data analysis was performed using GraphPad statistical software (GraphPad Software Inc., CA, USA), and IBM SPSS Statistics version 28 (IBM Inc., NY, USA). After initially recruiting only 7 males for the study, a decision was made to recruit more females for the study to both to improve applicability across sex and increase total sample size. One-way repeated measures ANOVAs were used to analyze the effect of bout on all outcome variables. The alpha level was set at 0.05. A Fisher's, least square difference, post-hoc test was used if a main effect was detected. Two-way repeated measures ANOVAs were used to analyze the interaction of trial and bout on all outcome variables. The alpha level was set at 0.05. A Bonferroni post-hoc analysis was used if a main effect for an interaction was detected. Normality was assessed by Shapiro-Wilk tests.

Previous studies reported the reliability of work-phase muscle oxygenation response (5, 10). As such, our reliability analysis used mean work SmO_2_ (%) as a primary outcome variable. Relative reliability was quantified as intraclass correlation coefficient (ICC_3,k_) (30, 31). Values of < 0.5 (poor), 0.5–75 (moderate), 0.75–0.9 (good), and > 0.9 (excellent) reliability were used (32). Absolute reliability evaluates the consistency of repeated measurements (repeatability) within a single individual. Absolute reliability was quantified as the standard error of the measurement (SEM) (30, 31, 33).

## 3. Results

### 3.1. Within session analysis

**Figure 3:**
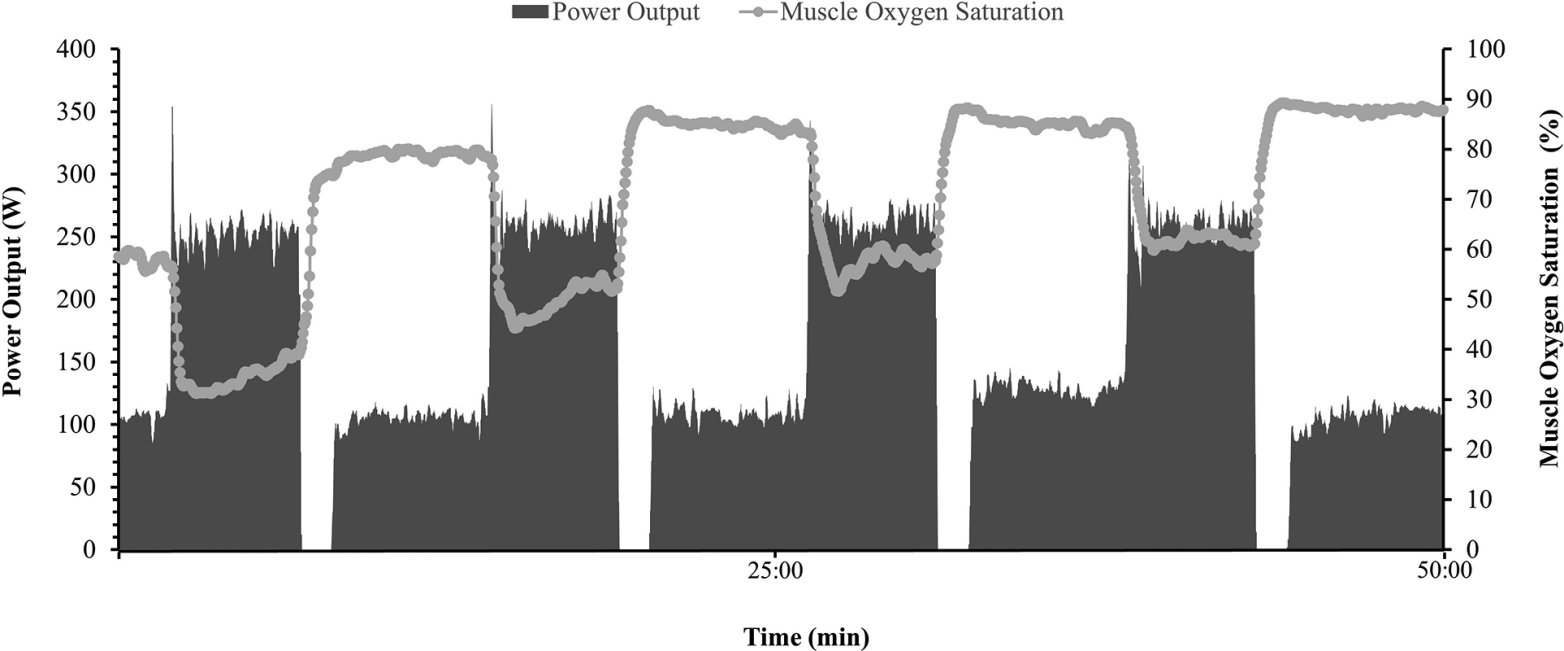
Representative dataset (*n* = 1) during a 50 min mixed intensity session. Power output (W) is represented on right Y axis by shaded bars, Heart rate (bpm) is presented on left Y axis by solid gray line, and muscle oxygen saturation (%) is displayed on left Y axis by gray dots and line.

**Figure 4:**
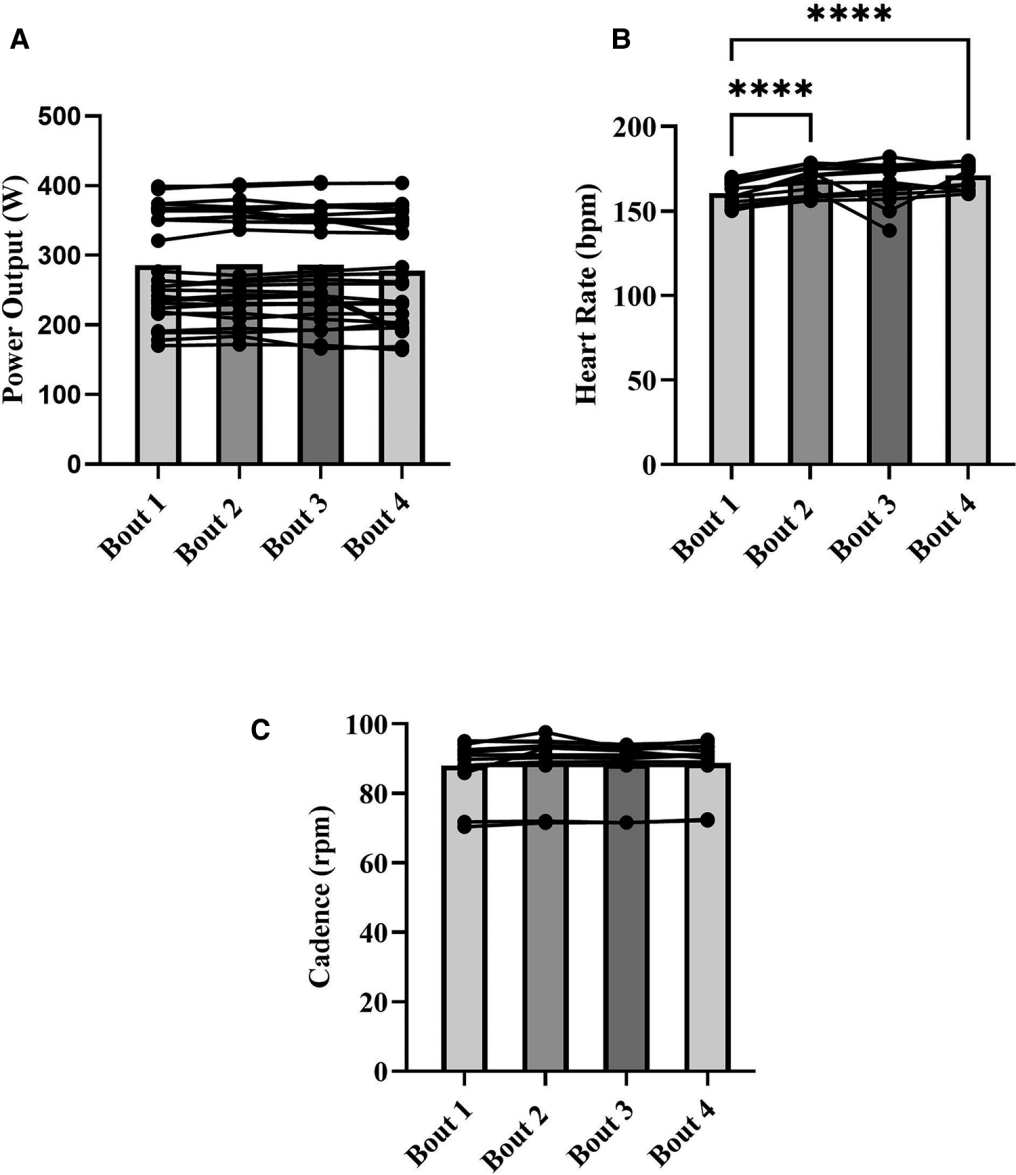
Means for power output (W) (**A**), heart rate (bpm) (**B**), and cadence (rpm) (**C**) for each severe intensity interval (bars), with representation of individual responses across the four bouts from both sessions (dots + lines). *p < 0.05, ****p < 0.0001.

Within session means for PO, HR, cadence, onset, work SmO_2_, and *Δ*SmO_2_ are presented in Table 1. A representative dataset for SmO_2_ during the severe intensity interval exercise is presented in Figure 3. For HR and cadence, data was available for only seven participants. One-way repeated measures ANOVAs were performed to detect an effect of bout on PO, HR, and cadence. No effect of bout on PO or cadence was detected (Figures 4A,C). A main effect of bout was found for HR (Figure 4B); mean HR at bouts 2 (168 ± 8 bpm), and 4 (170 ± 7 bpm) were significantly different from bout 1 (160 ± 6 bpm) (*p *< 0.05).

**Table 1:** Means for PO, HR, cadence, onset, work SmO_2_, and *Δ*SmO_2_ within session.

	BOUT 1	BOUT 2	BOUT 3	BOUT 4
Power (W)	282 ± 73	283 ± 73	282 ± 73	278 ± 76
Heart rate (bpm)^d^	160 ± 77	169 ± 8^b^	167 ± 12	171 ± 7^b^
Cadence (rpm)^d^	88 ± 88	89 ± 8	89 ± 8	89 ± 7
Onset SmO_2_ (%)	55 ± 15	58 ± 18	62 ± 17^c^	62 ± 18
Work SmO_2_ (%)	30 ± 12	32 ± 16	34 ± 18	37 ± 21^b^
*Δ*SmO_2_ (%)	27 ± 9	29 ± 10	31 ± 10	27 ± 10^a^

Data are presented in mean ± SD.

^a^ *p *< 0.05 from previous bout.

^b^ *p* < 0.05 from bout 1.

^c^ *p* < 0.05 between first two bouts and last two bouts.

^d^ *n* = 7.

**Figure 5:**
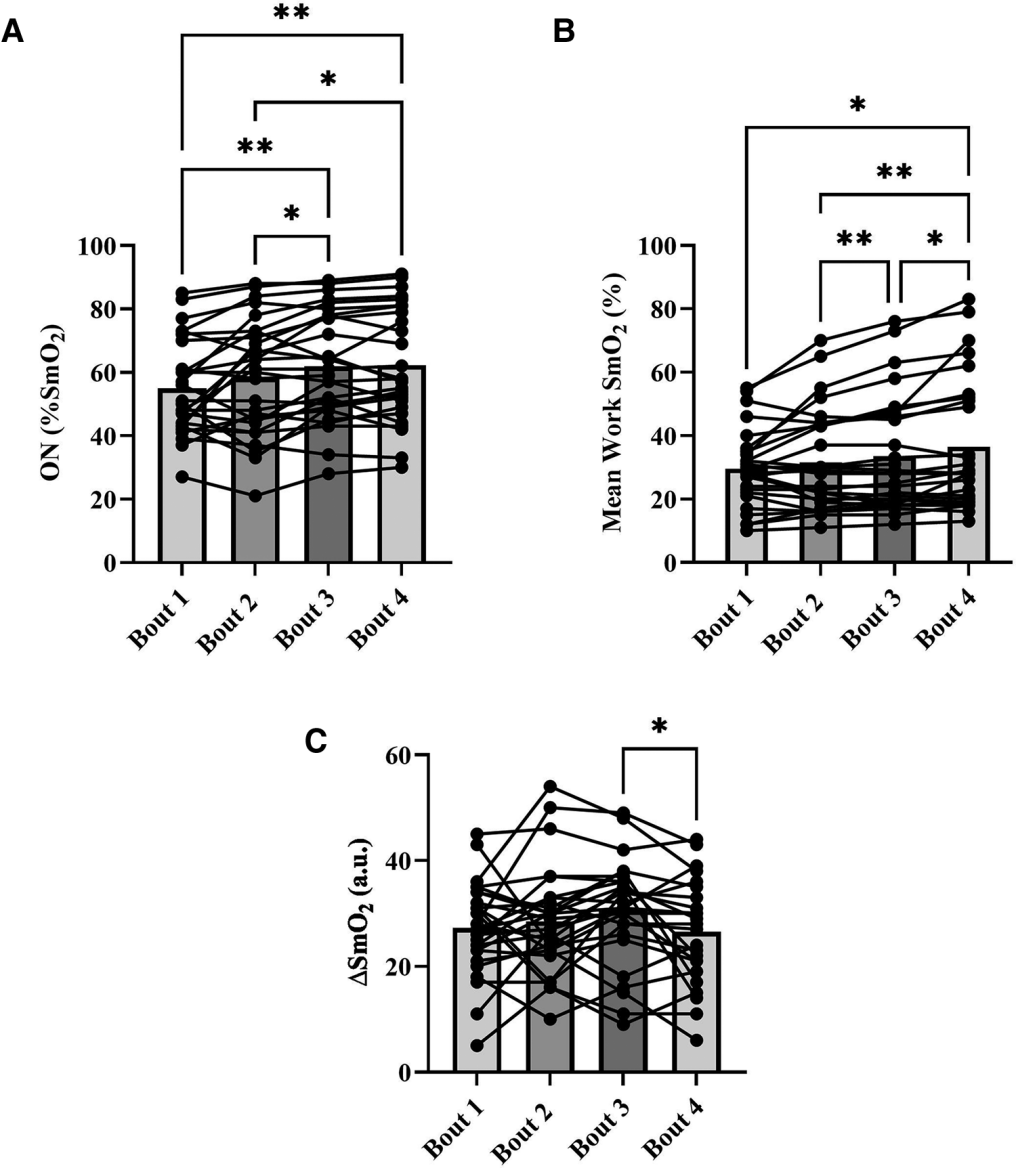
Means for muscle oxygen saturation (SmO_2_) at bout onset (%) (**A**), mean work SmO_2_ (%) (**B**), and mean SmO_2_ amplitude (*Δ*SmO_2_) (%) (**C**), for each severe intensity bout (bars), with representation of individual responses across the four bouts from both sessions (dots + lines). *p < 0.05, **p < 0.01.

For NIRS outcomes, one-way repeated measures ANOVAs were performed to detect an effect of bout on onset (%), work SmO_2_ (%), and *Δ*SmO_2_ (%). A main effect of bout was detected for all NIRS outcome variables. For onset, bout 1 (55 ± 15%) and 2 (58 ± 18%) were not different from each other but were different from bouts 3 (62 ± 17) and 4 (62 ± 18) (*p < *0.05) (Figure 5A). Significant differences in work SmO_2_ were detected across all bouts (*p < *0.05), with an increasing trend from 30 ± 12% in bout 1 to 37 ± 21% in bout 4 (Figure 5B). A significant difference was detected in *Δ*SmO_2_ between bout 3 (31 ± 10%) and bout 4 (27 ± 10%) (*p < *0.05) (Figure 5C).

### 3.2. Reliability analysis

Means for PO, HR, cadence, onset SmO_2_, work SmO_2_, and *Δ*SmO_2_, as well as relative and absolute agreements between sessions are presented in Table 2. Two-way repeated measures ANOVAs were used to detect a main effect for an interaction between trial and bout on all outcome variables. For PO, a main effect for an interaction was detected (*F* = 3.3, *p* < 0.05), with no main effect of either trial or bout. For cadence, no main effect for an interaction was detected, with no main effect of either trial or bout. For HR, a simple main effect of bout was found (*F* = 26.6, *p *< 0.01), with no main effect for an interaction, or for trial. For the effect of bout, a post-hoc analysis resulted in a significant difference between bouts 1 (161 ± 9, and 160 ± 8 bpm) and the remaining bouts (*p* < 0.05).

**Table 2:** Means for PO, HR, cadence, onset, work SmO_2_, and *Δ*SmO_2_ between sessions.

	SESSION 1	SESSION 2	ICC	SEM	95% CI
Power (W)	286 ± 75	283 ± 74	0.99	15	10–18
Heart rate (bpm)^a^	167 ± 9	168 ± 10	0.88	6	4–9
Cadence (rpm)^a^	89 ± 8	89 ± 7	0.98	2	2–3
Onset SmO_2_ (%)	58 ± 17	61 ± 17	0.77	17	13–23
Work SmO_2_ (%)	32 ± 17	34 ± 18	0.87	12	9–15
*Δ*SmO_2_ (%)	28 ± 9	29 ± 11	0.62	10	8–13

Data are presented in mean ± SD. Interclass correlation (ICC) present relative agreement between session. Standard error of the measurement (SEM) and 95% confidence intervals (CI) present absolute agreement.

^a^ *n* = 7.

For NIRS outcome variables, a simple main effect of bout on onset SmO_2_ was found (*F* = 6.2, *p* < 0.01), with no main effect for an interaction, or for trial. For the effect of bout, a post-hoc analysis resulted in a significant difference between bouts 1 (54.0 ± 16.0, and 56.0 ± 15.2%) and bouts 4 (62.3 ± 19, and 60 ± 19%) (*p* < 0.05). For work SmO_2_, a main effect for an interaction was detected (*F* = 4.2, *p* < 0.05). Simple main effects analysis showed that bout did have a statistically significant effect on work SmO_2_ (*F* = 5.5, *p* < 0.01). Post-hoc analysis detected a significant difference between bouts 2 (32.0 ± 18.0, and 29.1 ± 12.3%) and bouts 4 (36.0 ± 20, and 41.0 ± 23.3%) (*p* < 0.01). For *Δ*SmO_2_, no main effect for an interaction was detected, with no main effect of either trial or bout.

Lastly, repeated-measures relative reliability for mean work SmO_2_ showed good reliability (ICC = 0.87). The SEM for the same variable was 12% (CI 9%–15%).

## 4. Discussion

The aim of this study was to describe the effect of severe-intensity bouts on SmO_2_, measured by a wearable NIRS sensor at the VL muscle during self-paced cycling efforts, as well as to examine the reliability of SmO_2_ between sessions. Our first hypothesis was that SmO_2_ would desaturate with each bout number. Our results showed an effect of bout on SmO_2_ within session, but the effect was not as hypothesized. For our second aim, we hypothesized that SmO_2_ will demonstrate good reliability. No effect of trial was detected across all NIRS outcomes, and no difference was found between sessions, supporting our second hypothesis. Our protocol required each participant to sustain a pre-determined power target, estimated at 5% above their RCP PO, at a repeatable cadence and gear ratio.

From a subgroup analysis (*n* = 7), no difference in cadence was detected. For the same subgroup, HR response during bout 2 and 4 was significantly elevated relative to bout 1, suggesting a cardiac drift during exercise in the severe intensity domain that agrees with a prior report from Jones et al. (2011).Within each severe-intensity bout, muscle oxygenation displayed an expected desaturation response from the onset to the end of each bout, as seen in previous reports (16, 34–37). Our results showed an increase in onset SmO_2_ values between the first two bouts and final two bouts, reflecting post-exercise hyperemia, which is a function of metabolic disturbance (38–40). Additionally, work SmO_2_ significantly increased (i.e., less desaturation) between all bouts, with *Δ*SmO_2_ only presenting a significant decrease in amplitude between bouts 3 and 4. Contrary to our second hypothesis, both work SmO_2_ and *Δ*SmO_2_ demonstrated a resaturation response between bouts. Previous studies looking at SmO_2_ response during both an incremental exercise test, and constant-load exercise, present a continuous desaturation response to the limit of tolerance (14, 27, 37, 41, 42).

With sustained or repeated work bouts in the severe intensity domain, blood flow to working muscles will increase to try to match the demand for O_2_ as metabolites accumulate (43, 44). The increasing blood flow to the working muscle could be explained by accumulating metabolite concentration within the muscle (45). However, it may be hypothesized that O_2_ extraction would increase proportionate to O_2_ delivery, such that muscle V˙O_2_ would increase, i.e., responsible for the muscular contribution to the V˙O_2_ slow component during workload within the severe domain (16, 37, 46). Considering the other tissues detected by NIRS, blood flow may be expected to increase to the skin during work for evaporative heat loss. Skin and subcutaneous adipose tissue are less metabolically active, and so an increase in SmO_2_ would be expected for participants with higher skinfold thickness (9, 10, 47–49). This thermoregulatory cutaneous blood flow would also contribute a larger proportion to the overall NIRS signal (9, 48, 50, 51). As such, given the average skinfold measured at the VL in the current participant group (11.3 ± 5.4 mm), it is possible that the increasing SmO_2_ response between bouts is not related to changes within muscle, but rather due to non-muscle tissues under the wearable NIRS sensor.

In contrast to our results, Grassi et al. (2003), who investigated the effect of constant workload, high-intensity, cycling bouts on muscle oxygenation in the severe domain, found a continuous desaturation response during the bouts in conjunction with a continuous rise in pulmonary V˙O_2_ (36). This was further confirmed by Paquette et al. (2020), in trained sprint kayakers across three severe intensity bouts at different distances (200, 500, 1,000 m) using the same NIRS device as our study. We speculate the reason for the discrepancy with our results is due to the lower skinfold thickness reported for the trained kayakers (3.3 ± 0.4 mm) (41).

A study by Kriel, et al. (2018), reported no change in muscle oxygenation during repeated severe-intensity bouts, both in cycling and running exercise modes (23). This disagreement with our results is likely attributable to the difference in work duration spent in the severe domain in each bout. The cumulative time spent in the severe domain in Kriel, et al., was equal to 120 s, compared to 960 s used in this study. It is reasonable to assume that the changes observed in SmO_2_ response across bouts are related to the total duration or workload performed within the severe domain (16, 36, 37).

Lastly, with regards to test-retest reliability, our results showed that SmO_2_ measured by a wearable NIRS sensor was able to reliably indicate desaturation response in SmO_2_ during severe intensity self-paced cycling. Comparisons of the outcome variables between the two sessions showed no effect of trial. Relative and absolute reliability analysis for mean work SmO_2_ showed good reliability, corresponding to a SEM of ∼12%. Celie et al., reported similar ICC values (ICC = 0.87), when comparing forearm muscle contraction at 70% of maximal voluntary contraction (5). Other studies that measured the reliability of muscle oxygenation during whole-body incremental cycling exercise, reported higher ICC values at the VL (ICC = 0.94) (13). It is worth noting that both studies used a stationary, laser-based NIRS system (OxiplexTS, ISS, Champaign, USA). Also, their exercise protocol included resistance controlled by the ergometer (13). This is different from our self-paced efforts, during which the athlete was asked to match the gearing and cadence to sustain a specific target PO, rather than a fixed resistance set by the ergometer. The ecological nature of our exercise protocol as well as a higher accumulated fatigue, may account for the slightly lower reliability between sessions.

Considering these differences, our results highlight the practical capability of wearable NIRS sensors to provide a reliable test-retest measure of muscle oxygenation response under field-relevant training conditions. It is possible that practitioners can observe a hyperemic response during recovery between bouts, coupled with inhibition in SmO_2_ desaturation response during severe-intensity bouts, depending on the tissue composition under the wearable NIRS sensor. More work is needed to interpret the effect of tissue composition on this response prior to informing sport practitioners about how to design interval training based on muscle metabolic demand, in conjunction with subjective reporting from the athlete, heart rate data, and external load measures.

## 5. Limitations

Both HR and cadence were outcome variables of interest. As mentioned previously, measures of PO were measured in all participants, whereas cadence and HR were measured in only seven participants. These variables were not measured for the original sample, however, following a decision to recruit more females for the study, these variables were included as well. Despite the lack of data from the seven males, our sample size (*n* = 7) presented sufficient power (*β* = 0.08, *α* = 0.05) to eliminate a type two error for both HR and cadence (Supplementary Material). Skinfold measurements were obtained, but due to our sample size we were underpowered to analyze between group differences with skinfold as a main effect. Future studies should investigate the effect of body composition on muscle oxygenation from wearable NIRS during severe intensity exercise, as well as other intensity domains.

## 6. Conclusions

We conclude that SmO_2_ measurements, using a non-invasive, affordable, wearable NIRS sensor can demonstrate the heterogeneous muscle oxygenation response expected during severe intensity interval exercise on a cycling ergometer with good reliability between sessions in trained cyclists. The within session response presented an effect of bout number on SmO_2_ desaturation response. Despite hypothesizing SmO_2_ would desaturate with each bout, it responded in an opposite manner, demonstrating less desaturation with each bout. One explanation may be due to a significant contribution of non-muscular tissue to the O_2_HbMb signal. Despite finding good, between session reliability, sport practitioners should be aware that further investigation is needed to better understand the effect of severe intensity exercise on muscle oxygen saturation response using wearable NIRS, especially between groups with different body compositions.

## Data Availability

The original contributions presented in the study are included in the article/**Supplementary Material**, further inquiries can be directed to the corresponding author/s.
